# Bioinspired Stretchable MXene Deformation-Insensitive Hydrogel Temperature Sensors for Plant and Skin Electronics

**DOI:** 10.34133/research.0106

**Published:** 2023-06-02

**Authors:** Jun Wu, Yinghui Li, Shengshun Duan, Zhehan Wang, Xu Jing, Yucheng Lin, Di Zhu, Wei Lei, Qiongfeng Shi, Li Tao

**Affiliations:** ^1^ Joint International Research Laboratory of Information Display and Visualization, School of Electronic Science and Engineering, Southeast University, Nanjing, Jiangsu 210096, China.; ^2^ School of Materials Science and Engineering, Southeast University, Nanjing, Jiangsu 210096, China.; ^3^ Center of 2D Materials and Devices, Southeast University, Nanjing, Jiangsu 210096, China.

## Abstract

Temperature sensing is of high value in the wearable healthcare, robotics/prosthesis, and noncontact physiological monitoring. However, the common mechanic deformation, including pressing, bending, and stretching, usually causes undesirable feature size changes to the inner conductive network distribution of temperature sensors, which seriously influences the accuracy. Here, inspired by the transient receptor potential mechanism of biological thermoreceptors that could work precisely under various skin contortions, we propose an MXene/Clay/poly(N-isopropylacrylamide) (PNIPAM) (MCP) hydrogel with high stretchability, spike response, and deformation insensitivity. The dynamic spike response is triggered by the inner conductive network transformation from the 3-dimensional structure to the 2-dimensional surface after water being discharged at the threshold temperature. The water discharge is solely determined by the thermosensitivity of PNIPAM, which is free from mechanical deformation, so the MCP hydrogels can perform precise threshold temperature (32 °C) sensing under various deformation conditions, i.e., pressing and 15% stretching. As a proof of concept, we demonstrated the applications in plant electronics for the real-time surface temperature monitoring and skin electronics for communicating between human and machines. Our research opens venues for the accurate temperature-threshold sensation on the complicated surface and mechanical conditions.

## Introduction

Temperature sensors are vital to practical applications in smart healthcare, human–machine interfaces, robotics, and large-scale cultivation. However, for those sensors that are attached to the object surface, they are easily affected by the frequent movement of human and biorobots as well as the volume change of plants when they grow up. It is indispensable to render flexible temperature sensors with comparable stretchability to human skin or organic polymers, so it is highly desirable to develop temperature sensors that can function normally under stretch/pressure-induced deformations.

The sensor stability is susceptible to mechanical deformation because of the current mainstream temperature sensing mechanism, including the thermally sensitive resistor [1], the temperature-sensitive conductive polymers [2,3], pyroelectric materials [4], and the luminescent materials [5]. The temperature-induced changes of conductive network size or inner electron mobility are also coupled with mechanical deformation. For temperature sensors that rely on the percolation conductive network of thermistors [6–9], the size of capacitances [10], or the characteristic length of transistors [11,12], their output signals could also be affected by unexpected mechanical deformation. Hence, such undesired sensing characteristic tremendously complicates applications of flexible temperature sensors for quantitative measurements of temperature under varied pressure/stretch-induced deformations.

To decouple or eliminate the unwilling mechanical influence on the sensors, some preparatory work has been designed. One way to reduce this parasitic effect is to apply fractal design in layout, which is guided by the finite element method analysis of the mechanic [13,14]. However, this method only works effectively for the single-deformation case. Studies have also been focused on the lithographical patterning of the conducting constituents in mesh-like configurations, while this method typically involves complicated circuit designs [15,16]. Another way to avoid mechanical shocks is to utilize the photonic by combing thermo-optic effect and thermal expansion [17]. Although many photonic temperature sensors including waveguide Bragg gratings, PhC (Photonic Crystal Circular) nanobeam cavities show outstanding performance, they typically are composed of solid micromachines. Therefore, it is kind of complicated to realize them with flexible materials. Recently, researchers propose some precalibration procedures on the system level to decouple temperature from deformation [18–20]. However, such solutions require extra efforts on the integration and algorithm design that overcomplicate the sensing systems by requiring precalibration and even recalibration in runtime that sacrifices the sensing fidelity [19,20]. Therefore, on the sensor level, it remains challenging to propose a new temperature sensing mechanism decoupled from pressure/stretch-induced mechanical deformation (more comparison of our work to the reported ones could be found in Table S1).

Moreover, compared with continuous monitoring, there is a lack of study on the temperature-threshold sensors, which also play an important role in monitoring vital physiological information about the health condition, the machine functional mode, and the environmental state. The advantages are to reduce redundant temperature data and lower energy consumption. Similar to the warm and cold receptors in human skin, it is a viable solution to respond to critical threshold temperature that has the most important influence on the objective entities, such as the optimum growth temperature for the landscape plants, the livable temperature for the infants, and the healthy temperature for the immunocompromised patient.

Herein, inspired by the biological thermoreceptors from human, a hybrid MXene/Clay/poly(N-isopropylacrylamide) (PNIPAM) (MCP) hydrogel temperature sensor is fabricated with high stretchability and deformation-insensitive temperature sensing properties. The hydrophilic groups on the MXene enhance the lower critical solution temperature (LCST) of PNIPAM hydrogels. The resistance goes through a spike-like change during the heating, and the peak occurs at the LCST of hybrid hydrogels (~32 °C), which is proved to be insensitive to mechanical deformations. Inorganic clay substantially enhances the mechanical strength of PNIPAM hydrogels that could endure more than 100% stretching in 100 test cycles. The MXene nanosheets endow hydrogels with great electronic performance (0.01 S/m) and high thermal conductivity compared with pure PNIPAM hydrogels [21]. For proof of concept, the sensor is applied on the skin and the leaf to monitor the critical temperature threshold. A temperature sensor system composed of a flexible printed circuit board and MCP hydrogel sensor shows rich demonstrations as human–machine interfaces. The hydrogel-based temperature sensor can accurately distinguish temperature when the skin is stretched or pressed. Furthermore, the sensor applied on the leaf could accurately monitor the hot temperature even under the stretch. It can serve as a smart interface between humans, other biosome, and electronic devices by detecting the existence of the body and monitoring the critical temperature, which brings us one step forward toward the complex practical applicability of flexible temperature sensors.

## Results

**Fig. 1.**
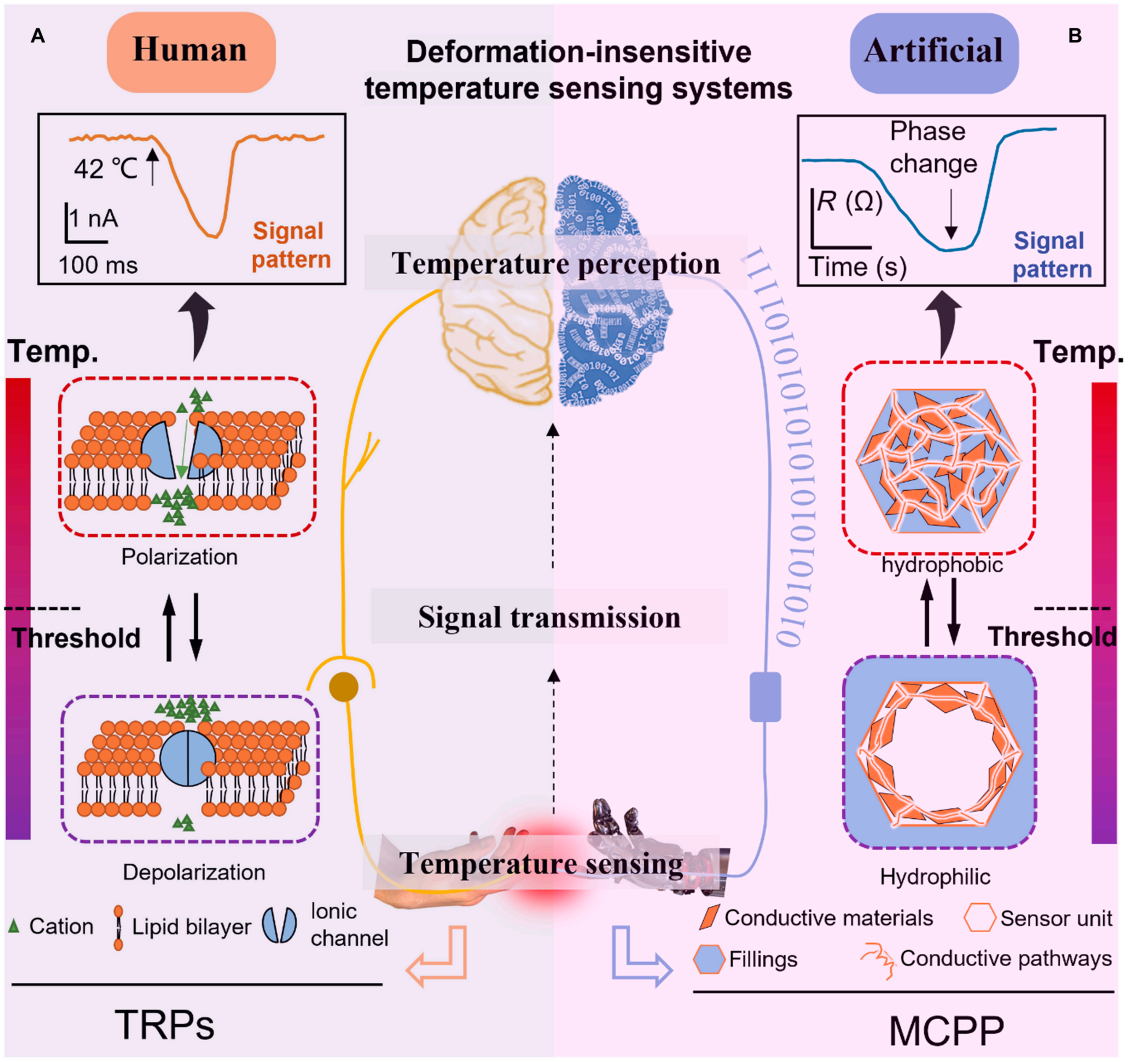
Temperature sensing mechanism for human body and the bioinspired model. (A) Human temperature detecting process. (B) The proposed temperature sensing model and process.

Human body temperature (Fig. 1A) can be detected precisely and continuously in biological tissues regardless of large-scale skin deformation by various stimulation, such as shear, pinch, and torsion [22–27], simply via temperature-induced ion transmission [28–30], which generates spike-responsive patterns when the temperature exceeds the threshold temperature. Such ion transmission mechanism and spike-responsive patterns are expected to provide a brand-new temperature sensing idea. Inspired by the transient receptor potential (TRP) of biological thermoreceptors, we propose a new temperature sensing principle on the basis of temperature-induced spike-responsive patterns, which could maintain accurate temperature measurement under mechanical deformation. As presented in Fig. 1B, at the lower temperature, the sensor cell is filled with supporting materials and conductive materials. When the temperature increases over a certain threshold value, fillings are discharged out of the cell the same as cations gets through ionic channels under activation temperature. Due to losing the support of fillings, inner conductive pathways would change and the whole resistance is altered. As seen, the biological sensing signal and electronic sensing signal of our bioinspired sensors are transmitted in the same form.

**Fig. 2.**
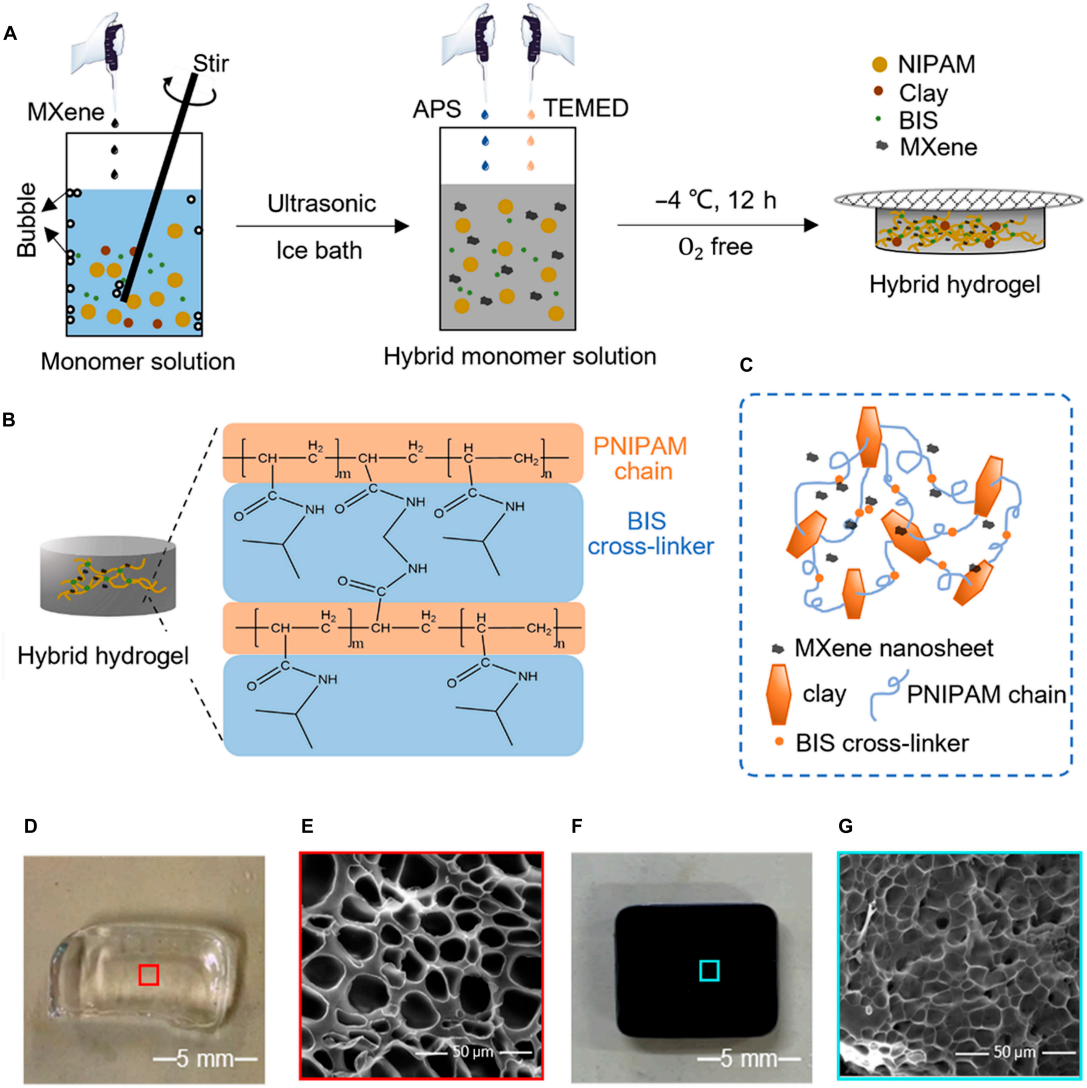
The material fabrication and characterization. (A) The fabrication process of MCP hydrogels through chemical and physical crosslinking. (B) The chemical crosslinking state of MCP hydrogels. (C) The physical crosslinking state of MCP hydrogels. (D) The physical picture of the PNIPAM hydrogel. (E) The SEM image of the PNIPAM hydrogel. (F) The physical picture of the MCP hydrogel. (G) The SEM image of the MCP hydrogel.

In our design, the thermosensitive PNIPAM hydrogel is chosen as the host unit. PNIPAM can exhibit significant characteristic change such as swelling/deswelling and absorption/desorption in response to the specific temperature; therefore, it is normally applied in actuators [31,32], smart drug delivery [33], and tissue engineering [34,35]. To improve its inherent mechanical weakness and enhance conductivity, inorganic clay and MXene nanosheets are chosen as the reinforced skeleton and conductive materials [36,37]. Figure 2A shows the one-step in situ polymerization process. Several abbreviations mentioned below are used to clearly distinguish hydrogels of different raw material content (Table S2). For example, “NC 0” represents that there is no clay content in the hybrid hydrogel; “NC 10”, “NC 5”, and “NC 3” represent that the mass ratio between N-isopropylacrylamide (NIPAM) and clay is 10:1, 5:1, and 3:1, respectively. The “MX 0” represents that there is no MXene content in the hybrid hydrogel; “MX10” and “MX40” represent that the MXene concentrations of hybrid hydrogels are 10 and 40 mg/ml, respectively (Table S2). As there are some bubbles containing oxide produced during stirring, which would disturb the polymerization process, the ultrasonic treatment and ice bath are applied to get rid of the oxygen and keep the solution cool to prevent NIPAM from self-aggregation. At a low temperature (4 °C), the accelerator N, N, N, N’-tetramethyl ethylenediamine (TEMED) and initiator ammonium peroxydisulfate (APS) collectively form a redox system which quickly initiates the polymerization.

Olefinic bonds of NIPAM and N, N′-methylenebis(acrylamide) (BIS) are connected to each other to form long polymer chains and to realize chemical crosslinking (Fig. 2B and S1) [38]. Traditional PNIPAM hydrogels mostly take organic crosslinkers BIS to be polymerized; however, such gels always exhibit very weak mechanical properties and are easily broken by applying external stresses [39]. Also, chemical-crosslinked gels exhibit slow deswelling behavior, often taking more than 3 d to approach equilibrium [40]. Here, we synthesized the inorganic clay nanosheets into the nanocomposite hydrogels, where the exfoliated inorganic clay serves as an effective multifunctional crosslinker (Fig. 2C). Apart from chemical molecular bonding, the PNIPAM chains also graft on the clay surface at one or both ends. The MCP hydrogels mainly consist of polymer chains connected by BIS and clay nanosheets. The pure PNIPAM hydrogel is transparent (Fig. 2D) and owns a typical 3-dimensional (3D) gel skeleton (Fig. 2E) from scanning electron microscopy (SEM).

Due to the oxygen and fluorine terminal groups on the surface (S1C), the MXene sheets own high hydrophily and could evenly disperse in the monomer solution to form steady hydrogels compared with carbon materials [41–44]. As shown in S2, the incorporation of MXene sheets decreases the transparency of hydrogels, but gels stay stable. For MCP hydrogels (Fig. 2F), it can be clearly found that the double crosslinking network provides far stronger, thicker, and continuous skeletons and less porosity that could afford more mechanical stimulus from SEM observations (Fig. 2G). The large distance between crosslinkers makes the polymer chains longer and more flexible, adopting more random conformations compared with hydrogels that are only crosslinked by BIS. Meanwhile, the physical crosslinking can also be prepared by in situ free-radical polymerization; therefore, the chemical and physical process can take place in the same solution.

**Fig. 3.**
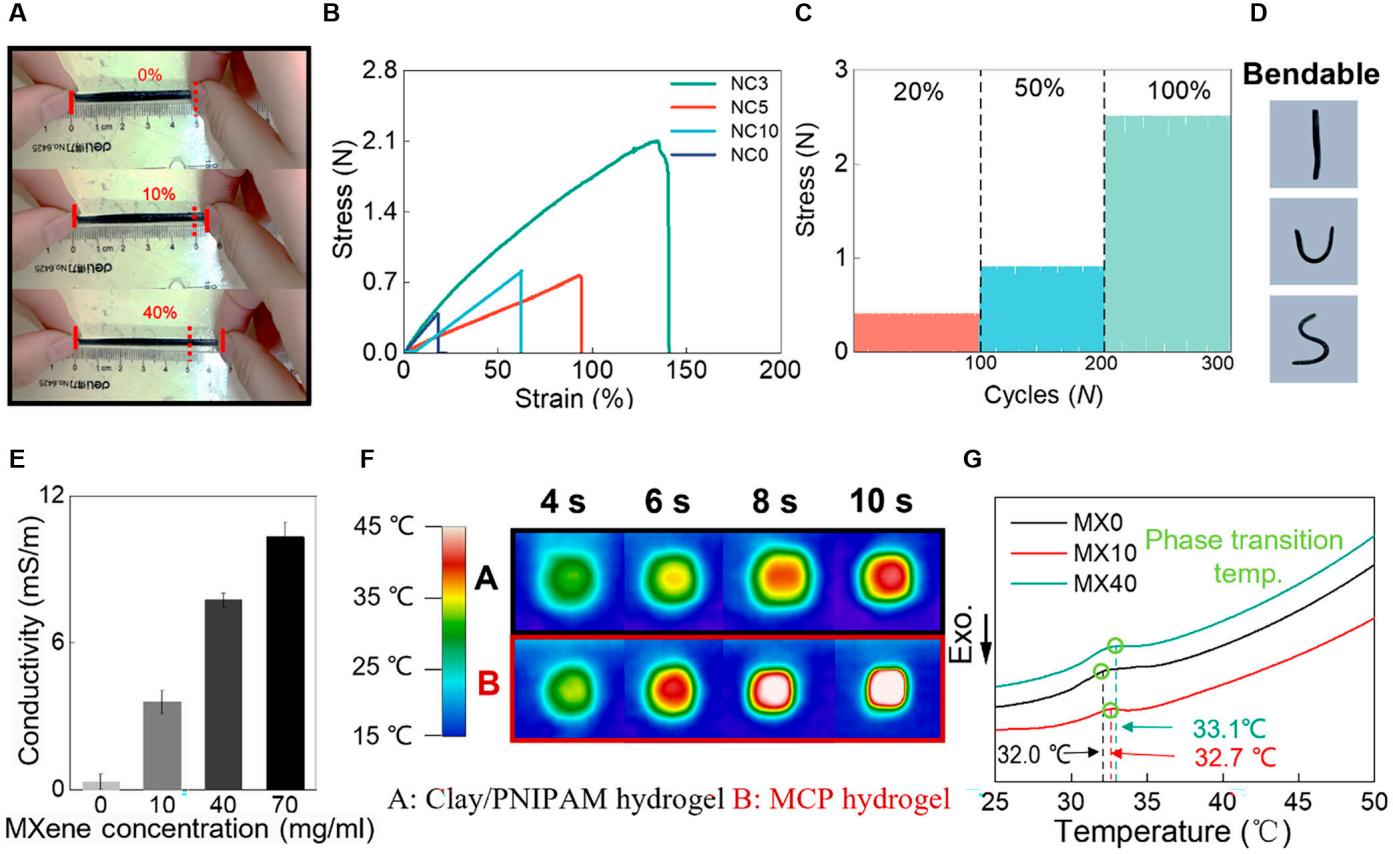
The function of adding inorganic clay and MXene nanosheets. (A) The hydrogel is stretched up to 10% and 40% of its original length. (B) The stress–strain tests on hybrid hydrogels of different clay content. (C) The periodical tests of 20%, 50%, and 100% stretching for 100 cycles. (D) The MCP hydrogels are bendable. (E) The electronic conductivity tests for hydrogels of different MXene solution content. (F) The thermal conductivity test under 50 °C. (G) The DSC tests of hydrogel samples MX0, MX10, and MX40.

The double crosslinking network highly enhances the stretchability of PNIPAM hydrogels. Therefore, the hydrogel sensor could conformally contact with human skin. Figure 3A shows that the MCP hydrogel can withstand large-scale deformations. After removing the external force, no damage is observed, and the original shape is restored. Figure 3B shows the stress–strain tests of a series of MCP hydrogels, respectively. As the clay content increases, the tensile strain increases and reaches a maximum elongation of 140%. For hydrogels of no clay content, they can only afford less than 20% strain. Furthermore, the tensile tests are investigated under 100 stretching–relaxing cycles at the low strain (20%) and high strain (100%). Results indicate that the MCP hydrogel has outstanding reliability at various levels of tensile strains (Fig. 3C). As shown in Fig. 3D, the MCP hydrogel is bendable and could be bent into various shapes.

Pure PNIPAM hydrogel owns poor electronic conductivity [38,45]. As shown in Fig. 3E, with increasing MXene concentration, the incorporation of an extra conductive network considerably enhances the electronic conductivity of hybrid hydrogels. In addition, the intrinsic thermal conductivity limits PNIPAM hydrogel applications in sensors that own low sensitivity and responsibility. Due to the large specific surface area, the MXene nanofillers can efficiently enhance the thermal properties of hydrogels [46–48]. The thermal diffusivity and conductivity performances are evaluated through infrared camera measurement. As shown in Fig. 3F, the pure Clay/PNIPAM hydrogel (denoted as “A”) and the MCP hydrogel (denoted as “B”) are placed on the hot plate that is set at a constant 45 °C, respectively. It can be observed that the MCP hydrogel increases its inner temperature faster than that of the Clay/PNIPAM hydrogel. After heating for about 6 s, the MCP hydrogel increases over 40 °C, while the pure one is still lower than 35 °C.

Furthermore, acrylamide-based polymers and some other polymers with amphiphilic character are known to exhibit an LCST property [49,50]. At temperatures below LCST, hydrogels absorb water to reach the swollen state and above LCST, they release their water. The temperature-induced phase transition in MCP hydrogels is investigated by differential scanning calorimetry (DSC) as shown in Fig. 3G. The measurements are done with the heating rate of 1 °C/min in the temperature range from 20 to 50 °C and under a nitrogen atmosphere. The endothermic peak for samples indicates a loss of weakly bound water molecules from the gel network that appears at LCST. The inorganic clay nanosheets seldom influence the Clay/PNIPAM hydrogels (S3). However, the addition of MXene slightly increases the LCST of MCP hydrogels from 32 to 33.1 °C, mainly resulting from the hydrophilic groups on the surface of MXene nanosheets that break the original balance between amphiphilic forces that comes from hydrophilic and hydrophobic groups of PNIPAM hydrogels.

The temperature sensing properties of MCP hydrogels are investigated by measuring and recording the temperature and real-time resistance, which is further presented by the relative resistance change (Δ*R*/*R*_0_). All hybrid hydrogels are mounted on a digital hot plate with a built-in temperature sensor and connected to a digital multimeter that is used to monitor electronic parameters. The real-time resistance and temperature data are captured and recorded at the same time by PC software on a laptop (S4). All samples are designed into the same dimension that is 20 mm in length, 5 mm in width, and 2 mm in thickness. Two copper wires of 0.8 mm in diameter are inserted into opposite sides of the samples as electrodes.

**Fig. 4.**
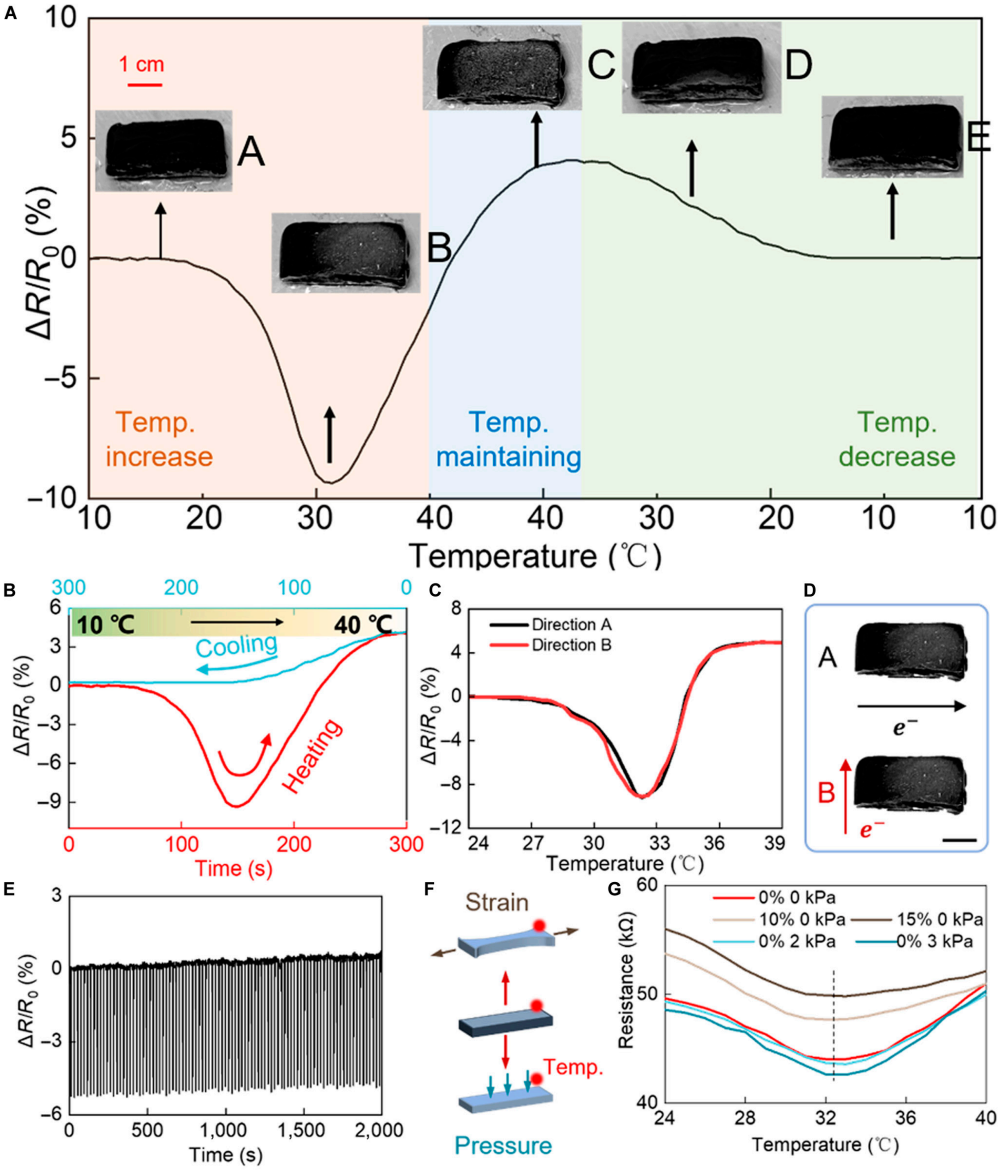
The electric performance of MCP hybrid hydrogels. (A)The resistance of hybrid hydrogels with temperature from 10 to 40 °C and naturally cooling down. (B) The resistance change difference between heating and cooling process. (C) The isotropic test on 2 orthorhombic directions. (D) The current direction. Scale bar: 1 cm. (E) Long-term durability of the MCP hydrogel under 100 cycles. (F) Schematic shows the applied mechanical stimuli. (G) The strain and pressure influence test.

Figure 4A illustrates the change in relative resistance with temperature. The plate first gradually rises to 40 °C and then is fixed at a constant 40 °C for a while in order that the hydrogel reaches the thermal equilibrium state. At last, it naturally cools down at room temperature. To clearly illustrate the resistance and the hydrogel state, here, the whole process is divided into 3 parts according to the temperature change in Fig. 4A. Inner pictures show a hybrid hydrogel sample respectively under 20 °C (picture A), 32 °C (picture B), 40 °C (picture C), 25 °C (cooling down, picture D), and 10 °C (cooling down, picture E) from left to right. At first, during the heating process, samples appear to have no obvious physical change and remain in soft gel states (picture A). As temperature increases (lower than 32 °C), the relative resistance begins to decrease. The external shape of the sample remains the same as the original until the temperature arrives at around 32 °C, which is recognized as the threshold temperature. Picture B shows the hydrogel when temperature increases over its threshold temperature. Parts of the gel become white, and several water droplets show on the surface of the gel. Along with more and more water molecules being excluded from the hybrid hydrogel, as in the constant-temperature (40 °C) part, the resistance begins increasing until the whole gel reaches thermal equilibrium with no more droplets appearing and the resistance seldomly changes. Gel in picture C almost turns white, and there are more water drops. After that, the hot plate is set to naturally cool down to room temperature. In this process, the water droplets are gradually absorbed into the gel with the resistance decreases and finally return to the original. Picture D shows the hydrogel when temperature decreases. The gel begins returning black and the water droplets on the surface begin to disappear. Picture F shows the hydrogel when temperature returns to the original. The state of hydrogel is similar to that in picture A. For different MXene concentrations, the temperature sensing properties of MCP hydrogels are further studied in S5. Similar to that in sample MX3, all the resistance curves go through the same trend. The turning threshold temperature increases a little with higher MXene concentration, which results from that MXene has influence on the LCST of hybrid hydrogels.

Usually for those metal temperature sensors or ceramic sensors, the linear resistance change between the heating process and cooling process is similar [20]. However, the temperature sensing mechanism largely depends on the phase change so that the change trend is not linear. We show a contrast curve in Fig. 4B to clearly distinguish the difference of hydrogels between the increasing and decreasing processes of temperature. During the heating process, the resistance goes through an LCST turning point due to the phase change and finally reaches the equilibrium state, while the cooling process does not go through the turning point compared with the heating process. Droplets that remained on the hydrogels are gradually reabsorbed into hydrogels, and gels become softer. Finally, the relative resistance returns to the original state. The existence of threshold temperature and related hydrogel state change causes such a nonlinear and unsymmetrical trend. The apparent variation between the heating and cooling process suggests that the temperature sensing mechanism of MCP hydrogels is different from traditional temperature sensors, which may provide a new technology for temperature trend detection.

For flexible and wearable devices, they usually need firmly conformal contact with complex human skin, meaning that multiple direction accesses are quite necessary [51]. Therefore, the demonstration of isotropic properties is measured by applying electrodes in different directions as shown in Fig. 4C and D. For the 2 perpendicular directions, the resistance changes stay in almost the same trend. Results demonstrate that MXene nanosheets are successfully dispersed uniformly based on our polymerization method, ensuring the isotropic property of hybrid hydrogels. The long-term durability test of the MCP hydrogel is studied under the heating (20 to 40 °C) and natural cooling processes (Fig. 4E). A robust signal output without any decay is observed during 100 cycles.

Conductive networks in soft elastic polymers are sensitive to mechanical deformation, usually caused by pressure, strain, and other mechanical stimuli. Wearable devices mounted on human skin frequently suffers from external pressing, leading to distortion in the system. For traditional temperature sensors fabricated with graphene, carbon nanotubes, and so on, the resistance is directly linked to temperature judgment [52]. The mechanical deformation results in strain–resistance drift problems that severely interferes with standard working conditions. As shown in S6, the resistance and related slope both increase as the sensor is stretched, meaning that such sensors cannot work accurately under large mechanical deformations.

According to previous research and the working mechanism of strain sensors, the strain could lengthen the conductive pathway and leads to an increase in the resistance. However, in our MCP sensors, before the temperature increases over the threshold, the resistance decreases due to the promotion of MXene conductivity. It is necessary to discuss the strain influence on the resistance change. Here, we test the resistance change from 0% to 10% of MCP hybrid hydrogels with strain from 0% to 25% (S7). To prove the deformation-insensitive property, the MCP hybrid hydrogel is stretched at strains of 5%, 10%, and 15% and pressed with 2 and 3kPa (Fig. 4F). In all the mechanic-stimulus tests, resistance changes in the same way when temperature increases and the threshold temperature all appears at around the LCST of hydrogels. The results indicate that MCP hydrogels enable deformation-insensitive temperature sensors, which can precisely detect threshold temperature even under mechanical stimuli like strain (Fig. 4G). Compared with the existing temperature sensors (Table S2), the MCP-hydrogel is insensitive to less than 25% strain stimulus and does not require complex readout circuit.

Here, we proposed 2-phase state models to describe the inner changes of MCP hybrid hydrogels during the temperature-rising process, which also explain the resistance change at threshold temperature, the resistance change trend, and the deformation-insensitive properties of hydrogels. Due to the coexistence of hydrophilic and hydrophobic side groups on hydrogel chains, the host matrix hydrogels, PNIPAM, are thermosensitive with significant phase change when the temperature reaches the LCST (~32 °C). Typically, when the temperature is below LCST, hydrophilic groups have strong hydrogen bonds with water molecules, resulting in a hydrophilic state where hydrogels are transparent, soft, and filled with water. To simplify the representation, we named it phase I. When the temperature rises over LCST, hydrogen bonds are broken by much stronger effects of hydrophobic groups, which leads to a hydrophobic state (phase II), where hydrogels gradually become white solid, along with water molecules being drained from hydrogels. As the direct result between hydrophilic groups and hydrophobic groups, LCST is independent of the mechanic state of PNIPAM, such as being pressed or stretched [53,54].

For conductive polymers and hydrogels, the conductivity highly relies on electronic network distribution. To explain the resistance performance of hybrid hydrogels, here, the inner morphologies and structures are further studied. At phase I, environmental scanning electron microscope (ESEM) is used to characterize the inner morphologies and structures of the hybrid hydrogel (S8A). Unlike SEM, which requires samples to be predried, hydrogels under ESEM check remain original inner microstructures full of water molecules. In the ESEM image of the hybrid hydrogel, the inner appears to be sponge-like porous structures, suggesting that MXene nanosheets have no obvious influence on the polymerization of hybrid hydrogels. As shown in S8B, these nanosheets conductively connect to each other from all aspects and naturally form a uniform 3D conductive network throughout the whole hydrogels.

However, in phase II, water molecules are excluded and the network collapses. The SEM image of freeze-dried samples (S8C) illustrates that without the support of water molecules, MXene nanosheets attach to the surface of PNIPAM hydrogel with a relatively loose arrangement, forming a bad electrical connection (S9). The original 3D conductive network is transformed into a 2D conductive surface with a possible theoretical model shown in S8D. As illustrated in S8E, and F, when the temperature rises over LCST of the hydrogels, the total number of conductive nodes and pathways quickly decreases due to dimensionality reduction. Therefore, from a macro point of view, there would be a sudden increase in resistance after LCST.

According to the phase state model, the electric performance and temperature sensitivity of MCP hybrid hydrogels can be explained as follows. As seen in Fig. 4A, there is no significant thermal effect on the hybrid hydrogels around ambient temperature, as the relative resistance barely changes. During the heating process, before the temperature rises to the LCST, hybrid hydrogels are hydrophilic and contain abundant water. Reasons for increases in electrical conductivity are attributed to metallic MXene nanosheets. Higher temperature can promote the conductivity of MXene because Ti_3_C_2_T*_X_* etched by the ceramic powder MAX (Ti_3_AlC_2_) belongs to transition metal carbides, which possess metallic negative TCR behavior, that is, the increase in temperature promotes charge carrier mobility [55].

When the temperature goes beyond the LCST of hybrid hydrogels, the hydrophilic to hydrophobic phase alternation results in spike response change. Along with more and more water excluding out of the network, resistance keeps increasing due to fewer conductive paths. When reaches the chemical bond balance, no more water droplets appear, and a stable resistance state is observed. However, during the naturally cooling process, water is reabsorbed into gels, so conductive networks recover from 2 to 3 dimensions. The cooling effect on MXene materials and hydrogels collectively leads to directly decreasing resistance (Fig. 4B). Compared to the TRP channel mechanism of biological thermoreceptors, the MCP sensor works in a similar way as the human temperature detecting process. The output signals change both results from the threshold temperature. The signal trend does not change until the temperature reaches its threshold. Over threshold temperature, the liquid inner hydrogel is discharged so the resistance begins increasing. This process is similar to that in receptor channels. Meanwhile, the threshold temperature also only relates to the intrinsic property of materials that cannot be influenced by the external mechanical deformation.

We then propose the hydrogel-based temperature sensor as a smart switcher interface for the human–machine interaction. Due to its response insensitivity to deformation, the sensor can accommodate skin deformation, which plays an important part in the human–machine interface and active touch switchers. Next, several experiments are performed to demonstrate the feasibility and potential applications of MCP hydrogel temperature sensors.

**Fig. 5.**
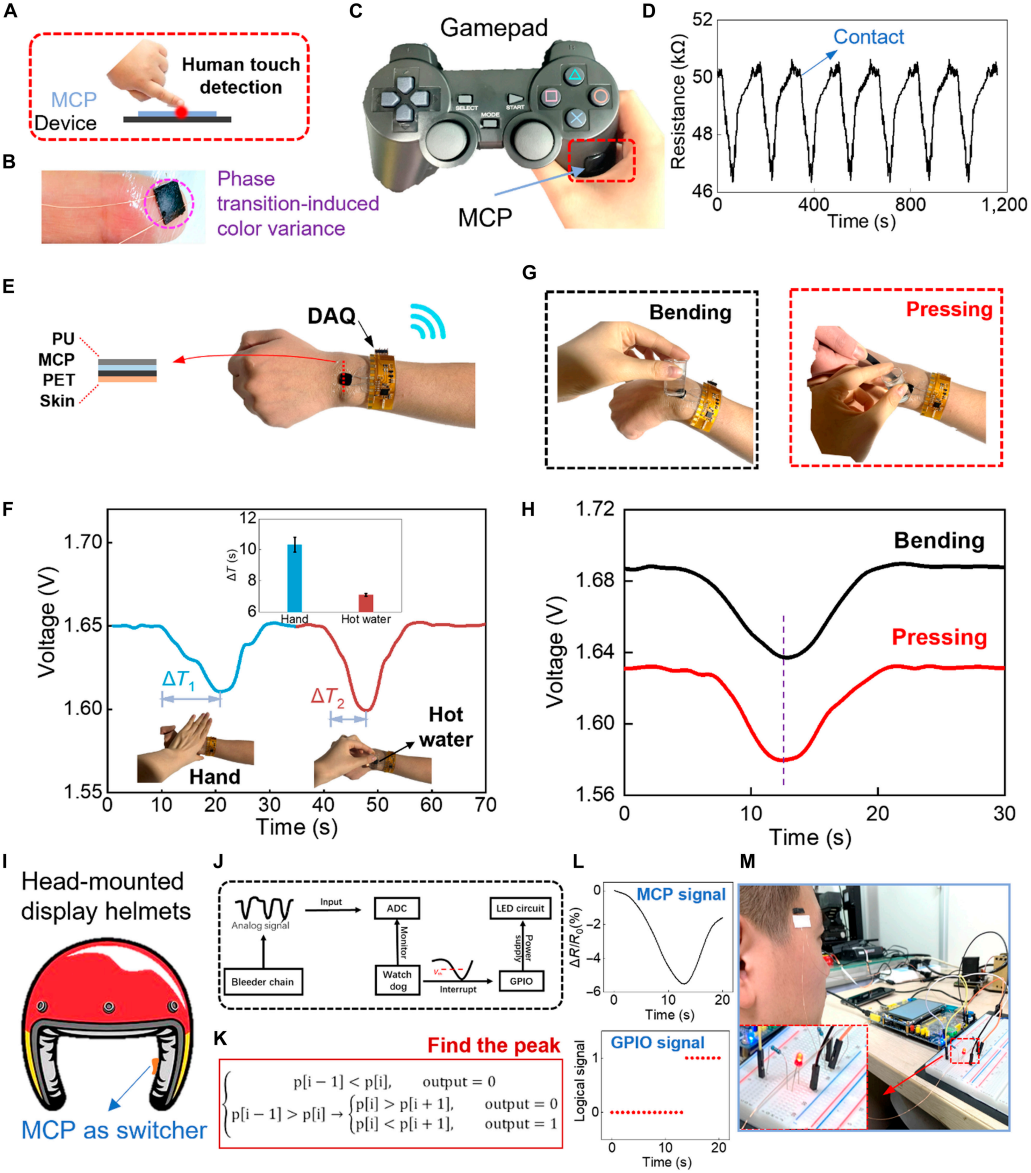
The MCP temperature sensor as an active human–machine switcher interface. (A) The MCP sensor could be attached on devices to detect human touch by temperature effect. (B) The forefinger touches the MCP hydrogel and induces a phase transition. (C) The MCP sensor was attached on a gamepad, as an active human–machine switcher. (D) The cyclic real-time channel signal of resistance response when a user touches the gamepad. (E) The temperature sensor module is mounted on the arm. (F) The output voltages of sensors touched by the palm and hot water. (G) Temperature sensing under skin being stretched and pressed condition. (H) Temperature sensing under skin being bended and pressed condition. (I) The MCP sensor could be used as a human–machine switcher for the head-mounted display helmets. (J) The corresponding circuit for the demonstration that detecting users and lighting a LED. (K) The algorithm that finding the peak. (L) The original signal and processed logical signal through the algorithm. (M) The demonstration that detecting users and lighting a LED. PET, polyethylene terephthalate; PU, polyurethane; GPIO, general-purpose input/output.

The MCP temperature sensor could be attached to any intelligent device to detect the human touch through the temperature effect of the human body (Fig. 5A). As the finger touches the MCP sensor (Fig. 5B), an evident phase change appears on the sensor with a color change from black to white. As a demonstration, the MCP sensor is attached to a gamepad, acting as an active human–machine switcher to detect human touch (Fig. 5C). The resistance response of the MCP sensor when a user touches the gamepad cyclically is depicted in Fig. 5D, exhibiting stable touch response capabilities. Besides, a wireless temperature sensor module is fabricated by a customized flexible printed circuit board and the sensor device. The sensor device consists of a polyethylene terephthalate substrate, MCP hydrogel, and polyurethane encapsulation layer. The flexible printed circuit board collects the analog signal from the sensor device and transmits it to a computer wirelessly (Fig. 5E). Figure 5F shows that the sensor module is mounted on the arm conformally, and it is touched by the palm and hot water (50 °C), respectively. The inner curve shows different changing time (Δ*T*) when the hydrogel reaches its threshold temperature. Apparently, the changing time for the hydrogel touched by hot water is largely shorter than that of the hydrogel touched by the palm, which probably results from the temperature of hot water being much higher than that of the human body. On that basis, the sensor applications under the skin state of being stretched and pressed by a glass rod are shown in Fig. 5G. The absolute values of output voltage (Fig. 5H) are slightly different due to the sensor resistance change, while they still show the same key turning trend to distinguish the temperature, which further proves the potential of deformation- unperturbed property of MCP hydrogel temperature sensors.

Furtherly, the MCP sensor can be used as the intelligent switcher for head-mounted display helmets (Fig. 5H). As a demonstration, we design a prototype that can detect the touch of the human body and make a response [lighting a light-emitting diode (LED) as an example], where the MCP sensor is combined with a microcontroller unit and a LED display circuit (S9). As shown in Fig. 5J, the analog-to-digital module of the microprocessor (STM32F104) captures real-time electronic signals from the MCP sensor, which are then processed by the clipping and filtering technique (S10) and a “find the peak” algorithm (Fig. 5K); when the watchdog module monitors a peak appearing, a high logical signal is generated and sent through the general-purpose input/output to light the LED. The MCP sensor signals and general-purpose input/output logical signals generated when the MCP touched the head of a user are shown in Fig. 5L, proving the precise peak detecting capabilities. The screenshots in Fig. 5K show that the LED is lighted when the MCP sensor is touched by the head of a user.

**Fig. 6.**
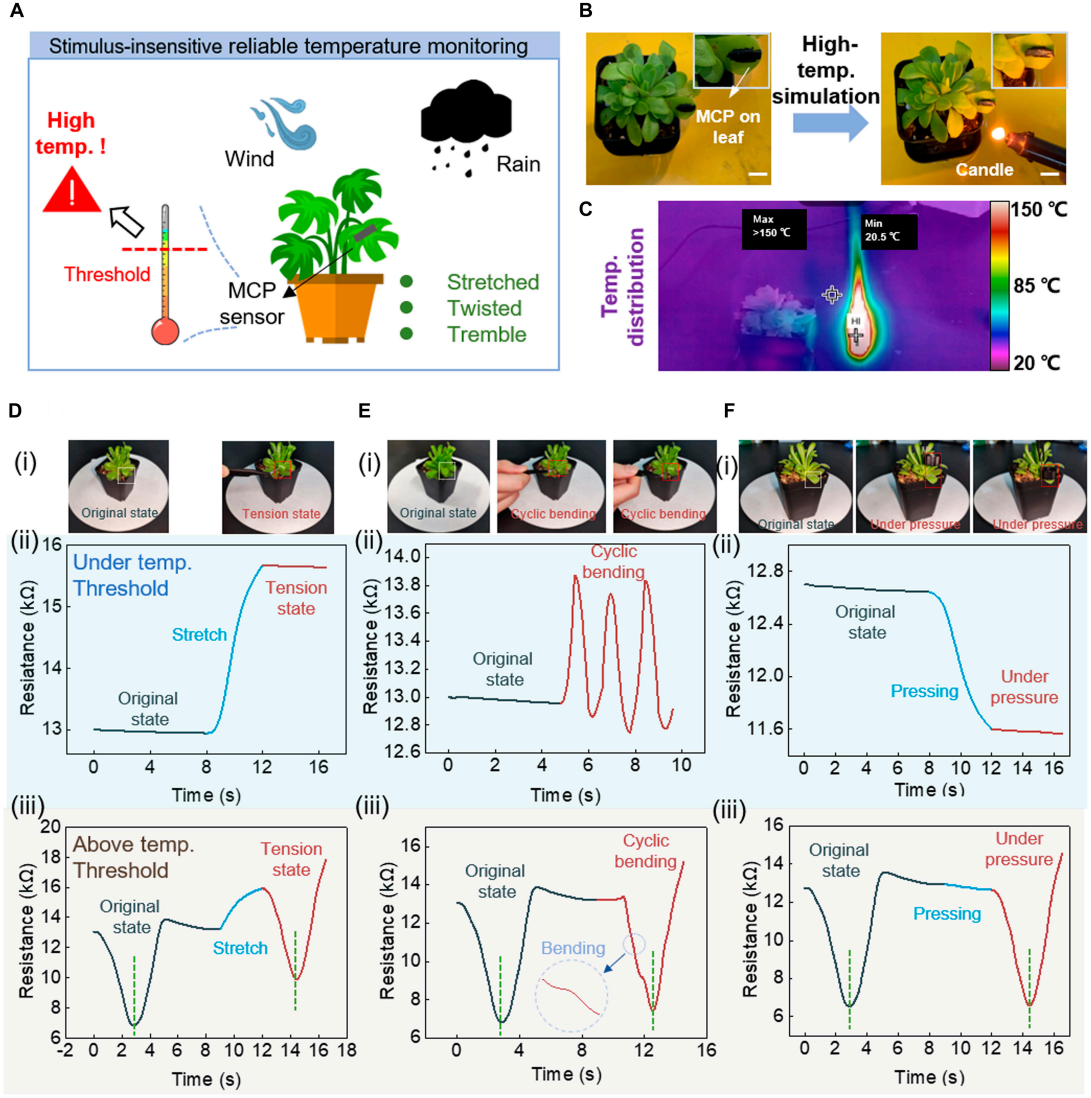
The MCP temperature sensor as a robust monitor for environmental temperature in plant electronics. (A) The concept of environment-free temperature monitoring application in plant electronics. (B) A candle is used to simulate the hot environment. (C) The infrared image showing the real-time temperature. (D to F) Precise temperature monitoring under 3 mechanical deformations: tension, cyclic bending, and pressure. (i) The photographs show the 3 types of mechanical stimuli. (ii) The temperature is lower than the threshold temperature (<32 °C). (iii) The temperature is lower than the threshold temperature (>32 °C).

For large-scale agricultural development, temperature greatly influences the appearance and productivity of plants. The plant growth has the lowest, most suitable, and maximum temperature ranges. On the one hand, for the crops, although the optimal temperature for growth is the temperature at which it grows the fastest, it is not the most robust temperature for plant growth. This is because at the optimal temperature, the organic matter in the plant is consumed too much, and the plant grows slender and weak. Therefore, in production practice, cultivating robust plants often requires a temperature lower than the optimal temperature, which is called the coordinated optimal temperature. On the other hand, for the ornamental crops, temperature will affect their flowering process and morphology, which are related to the economic value of plants. Therefore, the productive agricultural management requires innovative technologies to cope with complex growth environment. It is convenient to construct an Internet of Things-based plant growth temperature monitoring system that attaches the sensors to the surface of plants. For example, many plants (i.e., butterwort, onion, faba bean, lettuce, and cucumber) would grow abnormally or even die if the environmental temperature is above 32 °C. It is necessary to monitor the environment by the sensors attached to the surface of plants. Given the gradual growth-induced dynamic increase of plant size and environment-applied mechanical deformations (i.e., by wind, rain, or animals), the sensor should precisely monitor environmental temperature free from these mechanical deformations (Fig. 6A).

For a proof of concept, we place the MCP temperature sensor on butterwort leaves to monitor the environment temperature, and a candle is used to simulate the hot environment (>32 °C) that is unfriendly to the plants (Fig. 6B). The infrared image shows the temperature distribution around the butterwort when the candle is close to the butterwort (Fig. 6C). The 3 kinds of deformation stimuli are shown in Fig. 6Di, Ei, and Fi, including stretching, cyclic bending, and pressure. The output signals of the MCP sensor, under different mechanical stimuli when the environmental temperature is lower than 32 °C, are shown in Fig. 6Dii, Eii, and Fii. When the candle gets close to the butterwort, the environmental temperature around it would exceed the threshold temperature (32 °C). The sensor could accurately capture the change of temperature both in the original and in the 3 kinds of deformation states (Fig. 6Diii, Eiii, and Fiii), meaning that the MCP sensor can adapt to the plant growth and environment-induced mechanical deformations and can accurately monitor whether the temperature is above the threshold temperature.

## Discussion

A TRP-inspired MCP hybrid hydrogel has been proposed to realize deformation-insensitive temperature sensing. MXene and Clay are introduced into the hydrogel to enhance its electronic and thermal conductivity and reinforce strength. When the temperature rises over 32 °C, the transformation from a 3D network to a 2D plane results in significant resistance change. Rich demonstrations of the human–machine interaction application and smart plant electronics bring us one step forward toward practical applicability in human–machine interfaces and plant electronics, under the complicated surface or mechanical conditions. The MXene and hydrogel hybrid strategy could enable flexible temperature sensors with various thresholds by altering the LCST of hybrid hydrogels with additional material surface modifications, thus making it promising in hyperthermia patch fever detection and other temperature monitoring applications for wearable and plant electronics.

## Materials and Methods

### Materials

The Ti_3_C_2_T*_x_* (single layer, 98%) dispersion was purchased from 11 Technology Co., Ltd. The NIPAM (98%), N, N’-methylenebis (acrylamide) crosslinker (BIS, 99%), TEMED (98%), and APS (99%) were purchased from Aladdin company, and hectorite (Laponite XLG) was purchased from Nanocor company.

### Preparation of MXene/PNIPAM hybrid hydrogels

Ti_3_C_2_T*_x_* dispersions (0.5 ml; 5 mg/ml) were redispersed in 4.5 ml of deionized (DI) water by stirring with an ice water bath for 3 h. For other hybrid hydrogels of various MXene concentrations, the material ratios are listed in Table S2. Then, 500 mg of NIPAM monomers (recrystallization in hexane) and 17.5 mg of BIS crosslinkers were added with continuous stirring. The resulting solution was ultrasonicated for 10 min and purged with pure nitrogen to exclude bubbles and dissolved oxygen. After that, 10 μl of TEMED solution as the polymerization accelerator and 20 μl of APS (10 wt%) solution as the initiator were added successively. The uniformly dispersed solution was poured into a homemade mold and kept for 24 h at 4 °C. The obtained nanocomposite hydrogels were washed thoroughly with DI water to remove raw materials. The pure PNIPAM hydrogels were synthesized according to previous research.

### Preparation of MCP hybrid polymers

Ti_3_C_2_T*_x_* dispersions (0.5 ml; 5 mg/ml) were redispersed in 4.5 ml of DI water by stirring with an ice water bath for 3 h. For other hybrid hydrogels of various MXene concentrations, the material ratios were listed in Table S2. Then, 500 mg of NIPAM monomers (recrystallization in hexane), 100 mg of clay, and 17.5 mg of BIS crosslinkers were added with continuous stirring. The resulting solution was ultrasonicated for 10 min and purged with pure nitrogen to exclude bubble and dissolved oxygen. After that, 10 μl of TEMED solution as the polymerization accelerator and 20 μl of APS (10 wt%) solution as the initiator were added successively. The uniformly dispersed solution was poured into a homemade mold and kept for 24 h at 4 °C. The obtained nanocomposite hydrogels were washed thoroughly with DI water to remove raw materials.

### Device characterization

The structure, morphology, and component elements of the PNIPAM/MXene hybrid hydrogels were observed and recorded by a scanning electron microscope (Quanta 200 FEI), and ESEM (HITACHI). The temperature sensor is heated by a digital hot plate (Jingzhing, D20), with the electrical characterization of resistance change being measured by a digital multimeter (Keithley 2400). The DSC (PerkinElmer) is performed at the temperature ramp of 1 °C/min under a nitrogen atmosphere from 25 to 40 °C. The mechanic stretching test is performed by the force gauge (Pubtester, TST-01H). The real-time temperature detection is captured by infrared thermography (Fluke TiS20+).

## Data Availability

All the data needed to evaluate the conclusions in the paper are present in the paper and in the Supplementary Materials. Additional data related to this paper may be requested from the authors.
