# Supplementary material for: Bioinspired Stretchable MXene Deformation-Insensitive Hydrogel Temperature Sensors for Plant and Skin Electronics
Source: Research (Wash D C). 2023 Jun 2;6:0106. doi: 10.34133/research.0106 (PMC10237174; doi:10.34133/research.0106)
Supplement: Supplementary 1 — Tables S1 to S2. Figs. S1 to S10. [file research.0106.f1.docx]

Supporting Information

Bioinspired Stretchable MXene Deformation-insensitive Hydrogel Temperature Sensors for Plant and Skin Electronics

Jun Wu^1*^, Yinghui Li^1^, Shengshun Duan^1^, Zhehan Wang^2^, Xu Jing^2^, Yucheng Lin^1^, Di Zhu^1^, Wei Lei^1^, Qiongfeng Shi^1^, Li Tao^2,3^

^1^Joint International Research Laboratory of Information Display and Visualization, School of Electronic Science and Engineering, Southeast University, 210096, China

^2^School of Materials Science and Engineering, Southeast University

^3^Center of 2D Materials and Devices, Southeast University

**Table S1.** Summary of material ratios of MCP hydrogels.

| # | NIPAM  (mg) | Clay  (mg) | MXene (mg) | DI Water  (mL) | | BIS  (mg) | | 10% APS solution  (μL) | TEMED  (μL) |
| --- | --- | --- | --- | --- | --- | --- | --- | --- | --- |
| NC0 | 500 | 0 | 25 | | 5 | | 17.5 | 20 | 10 |
| NC3 | 500 | 167 | 25 | | 5 | | 17.5 | 20 | 10 |
| NC5 | 500 | 100 | 25 | | 5 | | 17.5 | 20 | 10 |
| NC10 | 500 | 50 | 25 | | 5 | | 17.5 | 20 | 10 |
| MX0 | 500 | 50 | 0 | | 5 | | 17.5 | 20 | 10 |
| MX10 | 500 | 50 | 50 | | 5 | | 17.5 | 20 | 10 |
| MX40 | 500 | 50 | 200 | | 5 | | 17.5 | 20 | 10 |

**Table S2.** Summary of material ratios of MCP hydrogels.

| Working mechanism | Signal mode | Materials | Mechanical stimulus sensitivity/Insensitive range | Circuit/Algorithm | | | Reference |
| --- | --- | --- | --- | --- | --- | --- | --- |
| Flexible Resistance Temperature Detector | Resistance | Flake graphite/Carbon nanotube/Polydimethylsiloxane | Sensitive | | Not required | ^1^ | |
| Flexible Resistance Temperature Detector | Resistance | CNT/PET:DOSS | 0~500Pa insensitive | | Not required | ^2^ | |
| Thermistor | Resistance | AgNW/PI | 0~100% strain insensitive | | Not required | ^3^ | |
| Thermistor | Resistance | Cr/Pt/PI | 0-60kPa | | A constant temperature difference circuit utilizes a Wheatstone bridge with a feedback control circuit. | ^4^ | |
| Thermistor | Resistance | Pt thin film | 0-1000Pa | | Not required | ^5^ | |
| Flexible Capacitance Temperature Detector | Capacitance | rGO/cellulose | Sensitive | | Readout circuit | ^6^ | |
| Thermocouple | Voltage | Bi-Te/Sb-Te | Sensitive | | Not required | ^7^ | |
| Thermochromic | Color | PVA/WPU/TC-M/NPCMs membranes | Sensitive | | Not required | ^8^ | |
| Temperature-threshold based sensor | Resistance | MXene/Clay/PNIPAM | 0-25%strain insensitive | | Not required | / | |


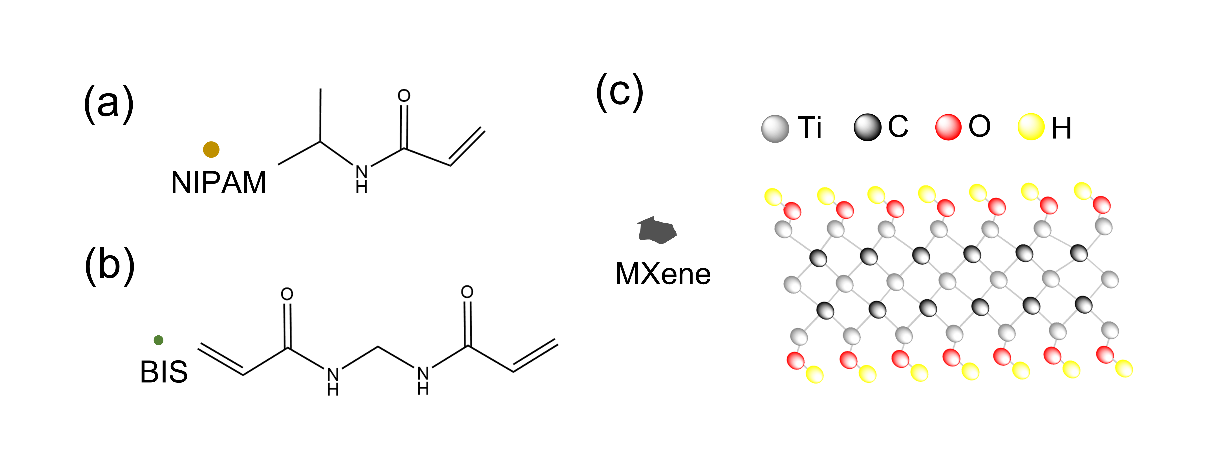


**Figure S1.** The molecular formula (a) NIPAM. (b)BIS. (c)MXene.


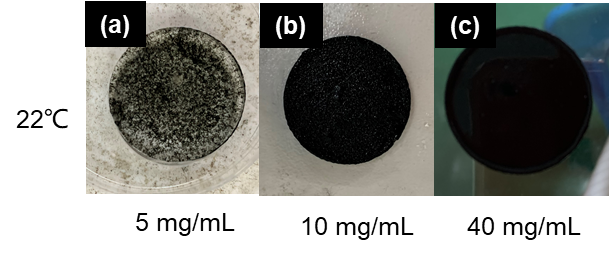


**Figure S2.** The appearance of MXene/PNIPAM hydrogels of different MXene concentration. (a) The MXene content is 5 mg/mL. (b) The MXene content is 10 mg/mL. (c) The MXene content is 40 mg/mL.

**Figure S3.** The DSC tests on Clay/PNIPAM hydrogels. The clay concentration of each hydrogel is 0, 2, 10, and 30 mg/mL.


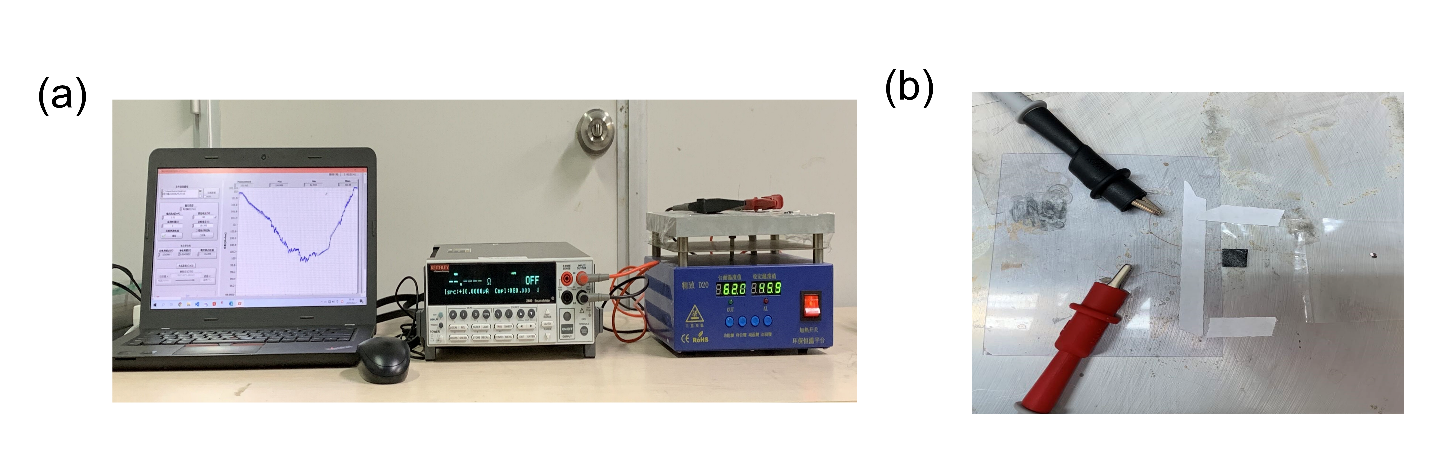


**Figure S4.** (a)The platform composed of the PC, Keithley 2400 digital multimeter and the hot plate. (b) The detailed test platform.

Resistance is measured by digital multimeter and temperature is detected by a built-in sensor of hot plate. Software on PC continuously collects and monitors the real-time resistance and temperature simultaneously.


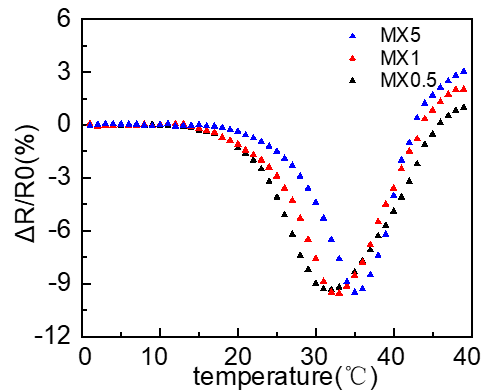


**Figure S5**. The temperature response of hydrogels with different MXene concentration.

**Figure S6.** The resistance of graphene sensors with temperature from 20 ℃to 60 ℃ and stretching to 0%, 10%, and 20%.

A home-made graphene temperature sensor is tested its stability under different stretching level. For temperature sensor highly relying on linear resistance response, resistance drift caused by conductive network deformation severely influences their normal work.

**
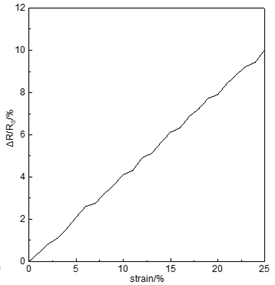
**

**Figure S7.** The resistance change with dynamic strain from 0% to 25%.

**
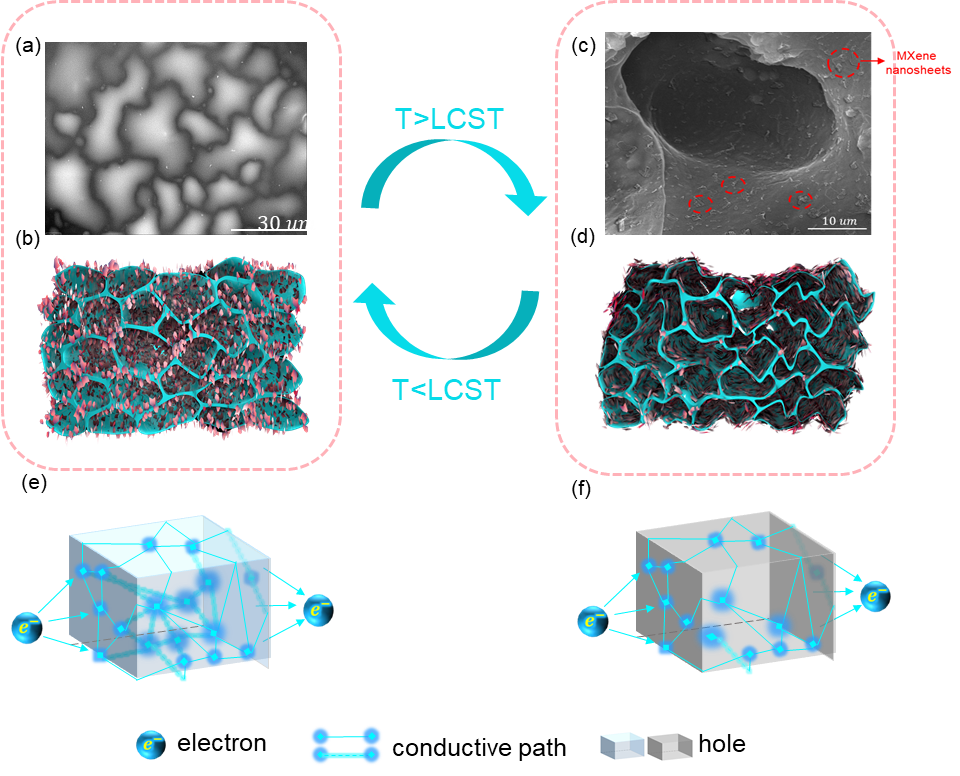
**

**Figure S8.** (a) The ESEM image of hybrid hydrogels. (b) The sketch of hybrid hydrogels before phase change. (c) The SEM image of hybrid hydrogels. (d) The sketch of hybrid hydrogels after phase change. (e-f) The simplified model to illustrate the conductive pathway change due to the phase change of hybrid hydrogels.


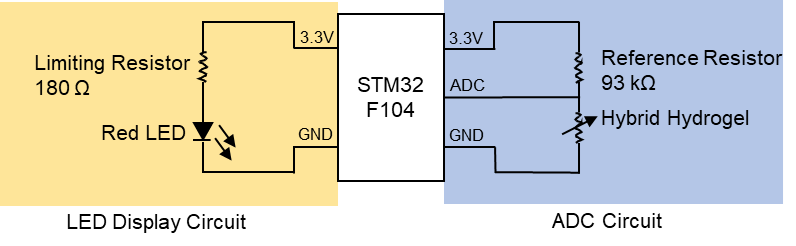


**Figure S9.** The LED circuit and logic control of the microcontroller.

The body temperature-sensitive conformal switch circuit combines microprocessor (STM32), resistors and light-emitting-diodes (LED), including the analog-to digital converting (ADC) part and the LED display part. The design fundamental depends on a bleeder chain circuits. STM32 output standard 3.3 V to series connection of a constant reference resistor (93 kΩ) and the hybrid hydrogel, while it captures and stores the voltages through the hydrogel by the ADC port at a frequency of 1 MHz, which is fast enough to judge the change of resistance. When the resistance of hybrid hydrogels decreases, the voltage would decrease as well. Meanwhile, the Watch Dog module in the system continuously compares the average voltage among ten sampling points for twice. Once the later average voltage is bigger than the former, the system would consider it as a resistance trending change and trigger the LED display circuit. When the display circuit is triggered, the port outputs standard 3.3 V to LED circuit. A protective limiting resistor (180 Ω) is used to avoid large current and short circuit.


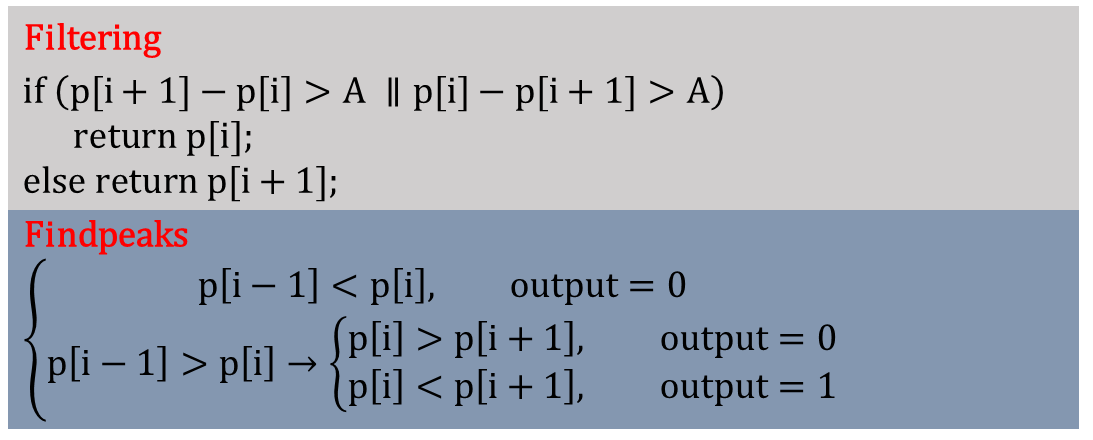


**Figure S10.** Clipping and Filtering Technique and ‘Findpeaks’ methods.

**References**

1.Wu, L.; Qian, J.; Peng, J.; Wang, K.; Liu, Z.; Ma, T.; Zhou, Y.; Wang, G.; Ye, S., Screen-printed flexible temperature sensor based on FG/CNT/PDMS composite with constant TCR. *Journal of Materials Science: Materials in Electronics* **2019,** *30*, 9593-9601.

2.Harada, S.; Honda, W.; Arie, T.; Akita, S.; Takei, K., Fully printed, highly sensitive multifunctional artificial electronic whisker arrays integrated with strain and temperature sensors. *ACS Nano* **2014,** *8* (4), 3921-3927.

3.Cui, Z.; Poblete, F. R.; Zhu, Y., Tailoring the temperature coefficient of resistance of silver nanowire nanocomposites and their application as stretchable temperature sensors. *ACS Appl. Mater. Interfaces* **2019,** *11* (19), 17836-17842.

4.Wang, L.; Zhu, R.; Li, G., Temperature and strain compensation for flexible sensors based on thermosensation. *ACS Appl. Mater. Interfaces* **2019,** *12* (1), 1953-1961.

5.Hua, Q.; Sun, J.; Liu, H.; Bao, R.; Yu, R.; Zhai, J.; Pan, C.; Wang, Z. L., Skin-inspired highly stretchable and conformable matrix networks for multifunctional sensing. *Nat. Commun.* **2018,** *9* (1), 244.

6.Sadasivuni, K. K.; Kafy, A.; Kim, H.-C.; Ko, H.-U.; Mun, S.; Kim, J., Reduced graphene oxide filled cellulose films for flexible temperature sensor application. *Synthetic Metals* **2015,** *206*, 154-161.

7.Lee, S. H.; Shen, H.; Han, S., Flexible thermoelectric module using Bi-Te and Sb-Te thin films for temperature sensors. *Journal of Electronic Materials* **2019,** *48*, 5464-5470.

8.He, Y.; Li, W.; Han, N.; Wang, J.; Zhang, X., Facile flexible reversible thermochromic membranes based on micro/nanoencapsulated phase change materials for wearable temperature sensor. *Applied energy* **2019,** *247*, 615-629.
